# DNA–protein interaction studies: a historical and comparative analysis

**DOI:** 10.1186/s13007-021-00780-z

**Published:** 2021-07-23

**Authors:** Ricardo André Campos Ferraz, Ana Lúcia Gonçalves Lopes, Jessy Ariana Faria da Silva, Diana Filipa Viana Moreira, Maria João Nogueira Ferreira, Sílvia Vieira de Almeida Coimbra

**Affiliations:** 1 Departamento de Biologia, Faculdade de Ciências da Universidade do Porto, Porto, Portugal; 2 LAQV Requimte, Sustainable Chemistry, Universidade do Porto, Porto, Portugal; 3 Universidade do Minho, Braga, Portugal

**Keywords:** ChIP, DNA-footprinting, DNA–protein interaction, EMSA, SELEX, SPR

## Abstract

DNA–protein interactions are essential for several molecular and cellular mechanisms, such as transcription, transcriptional regulation, DNA modifications, among others. For many decades scientists tried to unravel how DNA links to proteins, forming complex and vital interactions. However, the high number of techniques developed for the study of these interactions made the choice of the appropriate technique a difficult task. This review intends to provide a historical context and compile the methods that describe DNA–protein interactions according to the purpose of each approach, summarise the respective advantages and disadvantages and give some examples of recent uses for each technique. The final aim of this work is to help in deciding which technique to perform according to the objectives and capacities of each research team. Considering the DNA–binding proteins characterisation, filter binding assay and EMSA are easy in vitro methods that rapidly identify nucleic acid-protein binding interactions. To find DNA-binding sites, DNA-footprinting is indeed an easier, faster and reliable approach, however, techniques involving base analogues and base-site selection are more precise. Concerning binding kinetics and affinities, filter binding assay and EMSA are useful and easy methods, although SPR and spectroscopy techniques are more sensitive. Finally, relatively to genome-wide studies, ChIP–seq is the desired method, given the coverage and resolution of the technique. In conclusion, although some experiments are easier and faster than others, when designing a DNA–protein interaction study several concerns should be taken and different techniques may need to be considered, since different methods confer different precisions and accuracies.

## Introduction

It has been known since the second half of the last century that the binding of a protein to a DNA molecule has a very important role in the function of a living cell and in life’s sustainability itself. For many decades and representing a big segment of the molecular biology research conducted, scientists tried to unravel how DNA links to proteins, forming complex and vital interactions. In the early beginning of these studies, even before the publication of the DNA molecular structure, Stedman and Stedman [1] already referred to histones as potential regulators of the DNA biological activity. Since then, scientists have not abandoned this research field, having unravelled many details about the crucial interaction between proteins and DNA. This interaction is responsible for essential molecular and cellular mechanisms, such as transcription, transcriptional regulation, recombination, replication, DNA repair, viral infection, DNA packing and DNA modifications [2]. The studies usually performed were either from a purely chemical perspective, analysing the structure of the complex formed, or from a transcriptomic level, investigating if a certain protein does bind to a particular DNA or gene and the interference of this interaction in gene expression, existing great intersection between both approaches [3].

From a molecular point of view, lifting the veil from the way a DNA molecule binds to a protein and starting to distinguish some patterns and possible favoured interactions between amino acids and DNA base sequences, in the sixties Leng and Felsenfeld [4] discovered that polylysine polypeptides interact preferably with A-T-rich DNA, while polyarginine connects desirably to G-C-rich DNA. A decade later, Seeman et al. [5] shed a little more light upon the structure of these interactions and, using the hydrogen-bonding atoms identified by these researchers on DNA base edges, suggested that specific amino acid side chains recognise certain nucleotides and that there is an increased likelihood that these interactions are more specific in the DNA major groove than in the minor groove. Indeed, a few years later, the model building studies that resulted from the McKay and Steitz [6] pioneer detection of a DNA–protein complex using X-ray crystallography suggested that *Escherichia* *coli* catabolite gene activator protein (CAP) does bind to the DNA major groove. Later, Pabo and Sauer [7] and Matthews [8] continued to study the amino acid–base connection and included electrostatic and van der Waals interactions in the models developed. Nowadays, despite knowing that there are some preferred interactions, it is accepted that protein families bind to DNA in different ways depending on the complex formed, not existing a straightforward correspondence between amino acids and bases of nucleic acids [9]. The majority of solely chemical investigations related to DNA–protein binding performed more recently focus on a specific interaction and the techniques used in this context enable to collect data related to several aspects such as the protein’s size, the DNA-binding site, the strength of the binding, the effects of the protein binding on the structure of the DNA and individual groups or specific bases involved in the interaction [3].

As for the study of transcription factors (TFs) and the regulation of gene expression, it was also in the sixties that François Jacob and Jacques Monod discovered the genetic regulatory mechanism in prokaryotes moderated by the *lac* operon [10]. Many findings followed that managed to decipher the mechanism that controls gene expression. Generally, the studies that use these techniques are centred on a specific protein, somehow related to the phenotype being investigated, which is suspected of binding to the promoter region of a potential target gene. The aim of each technique may be to recognise DNA-binding proteins in a cell extract, identify the DNA-binding site, analyse the specificity of the binding, or simply confirm if a given protein does bind to the respective alleged target genes and determine the effects of the binding in these genes’ expression, envisioning to reveal novel insights into gene regulatory systems [3].

This review intends to compile and briefly describe the majority of the existing techniques, and the respective variants, that enable to access information related to DNA–protein interactions, trying to combine them according to their purpose, knowing that there is an overlap between certain methods. The positive and negative aspects, as well as some improvements and slight modifications performed to each procedure referred in recent studies will also be noted. The final aim of this article is to aid researchers in the moment of deciding which technique to perform according to the objectives and capacities of each research team, hoping to contribute to the increase of the current knowledge related to DNA–protein interactions and gene regulatory networks.

## Studying DNA–protein interactions

### DNA-binding proteins characterisation

#### Filter binding assay and electrophoretic mobility shift assay (EMSA)

**Fig. 1:**
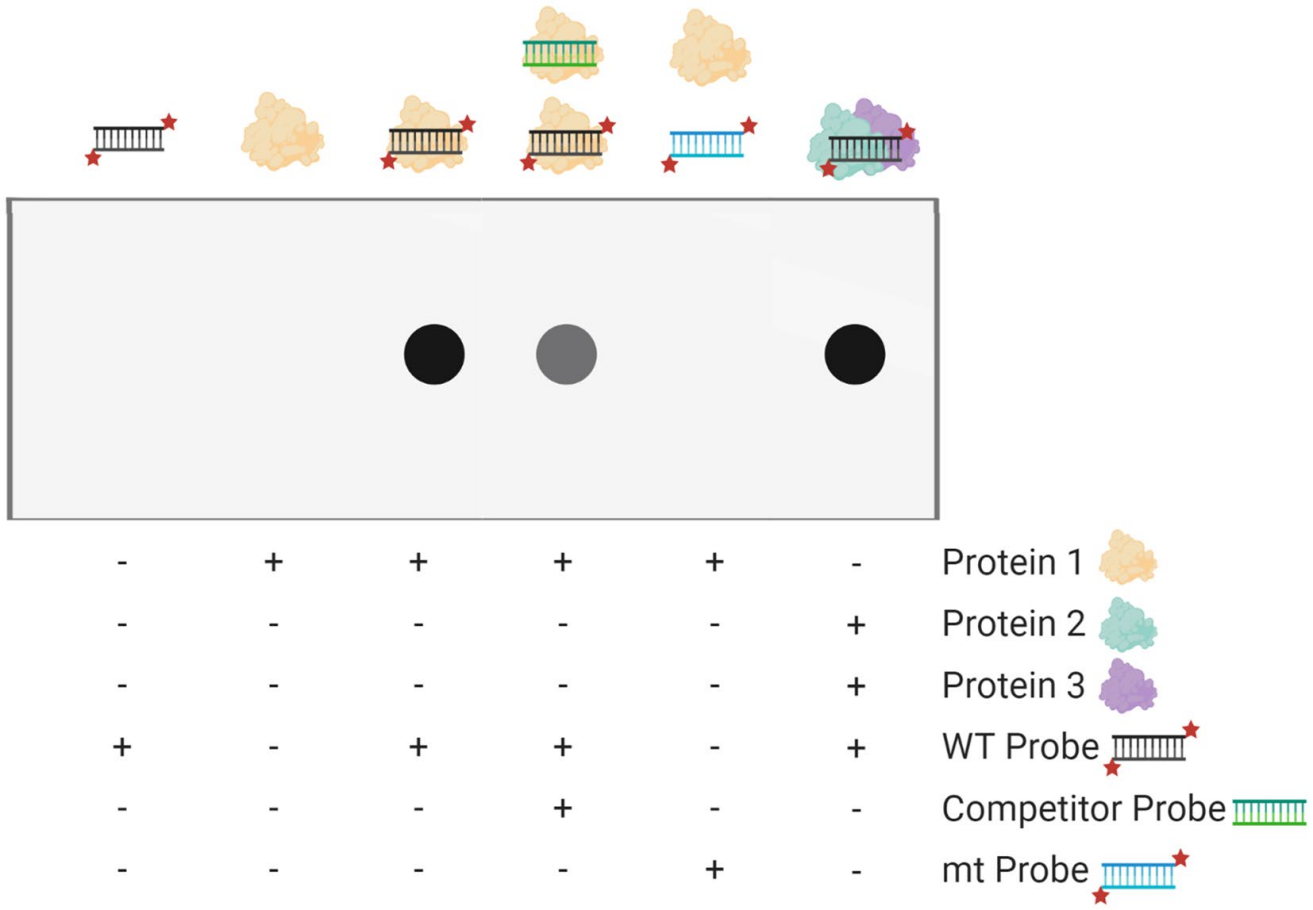
Representation of the expected results of a filter binding assay visualised in a nitrocellulose membrane. While in the first two lanes, no signal is detected, since only one of the complex molecules is present, labelled nucleic acid (WT probe, first lane) and protein of interest (protein 1, second lane), in the third lane, the complex is formed and the signal is detected, since the protein, that is attached to nitrocellulose membrane, is linked to a labelled nucleic acid. In the forth lane, an unlabelled competitor probe deviates some proteins to the formation of a different complex, leading to the weakening of the signal. In the fifth lane, the substitution of the WT probe by a labelled mutated fragment (mt probe) enables the formation of the complex, in case the mutation affects the complex formation. Finally, the last lane represents the formation of a new complex involving the WT labelled probe and two different proteins, leading to a signal quite similar to the one observed in lane 3. Created with BioRender.com

The interaction between nucleic acids and proteins was not yet totally described when Yarus and Berg [11] developed the filter binding assay that relies in the fact that a major part of proteins can be retained in a nitrocellulose membrane. In case the protein under study does bind to a nucleic acid, subsequently the complex may also be held in the nitrocellulose filter. This method is quite inexpensive, simple and relatively rapid, starts by the extraction and purification of the protein of interest and radio-labelling of the nucleic acid, followed by the binding reaction and ends in the filtering technique, which consists in applying vacuum to a porous plastic disc placed bellow a nitrocellulose filter that is impregnated with a solution containing the binding reaction. Then, the results are revealed and the quantities of bound nucleic acid are calculated using a phosphorimager [12] (Fig. 1).

Membrane filters were already being used for some time to detect binding between molecules [13], when Jones and Berg [14] tested these membranes to study the interaction between DNA and proteins. However, this last experiment was a bit dubious since the protein itself would not bind to the membrane. It was around this time that Yarus and Berg [11] introduced the nitrocellulose membranes to verify the recognition of a tRNA by an enzyme and, just a year later, Riggs et al. [15] continued improving this method by expanding its applicability, managing to properly detect the binding of a protein to DNA, more specifically the *lac* repressor-operator interaction. This new approach replaced the tedious and limited techniques that were being used until then, such as glycerol or sucrose density gradients and DNA columns.

However, this procedure presents several drawbacks that have led to its disuse. Firstly, not all the proteins bind to nitrocellulose membranes and some even denature when clinging to these filters. Moreover, if the interaction between the molecules of the complex is not strong enough, it may not withstand the filtration process [16]. Furthermore, using this method one cannot recover and analyse the composition of the binding reaction resulting products, making it impossible to determine if a DNA molecule binds to more than one protein, since the DNA only needs to interact to a single protein in order to be retained in the filter [17] (Fig. 1). Additionally, if single-stranded nucleic acids adhere randomly to the filter, which happens under certain solution conditions, it may result in undesirable interferences that might obscure the true binding signal [18].

Finally, a technique was developed that uses gel electrophoresis and that surpasses these inconveniences. Fried and Crothers [17] and Garner and Revzin [19] created the electrophoretic mobility shift assay (EMSA), which consists in evaluating if a protein causes a retardation in the electrophoretic run of a nucleic acid fragment when bound to it in a complex compared to the run of the same nucleic acid not bound to a protein. Like the filter binding assay mentioned above, EMSA tests the nucleic acid–protein interaction qualitatively, but unlike the first one, EMSA also does it quantitatively, since the mobility of the nucleic acid fragment in the gel decreases as the number of proteins bound to it increases. So, this method assesses not only the stoichiometric ratio of protein linked to the nucleic acid, but also the relative binding affinities of a certain protein for two different nucleic acids [17]. Furthermore, if, in the beginning of the electrophoresis, the nucleic acid fragments that are not involved in any interaction enter the electrophoretic gel before the dissociation of the complexes formed, it is also possible to quantify the concentration of unbound nucleic acid fragments and, thus, the concentration of the complexes in the binding reaction [19].

**Fig. 2:**
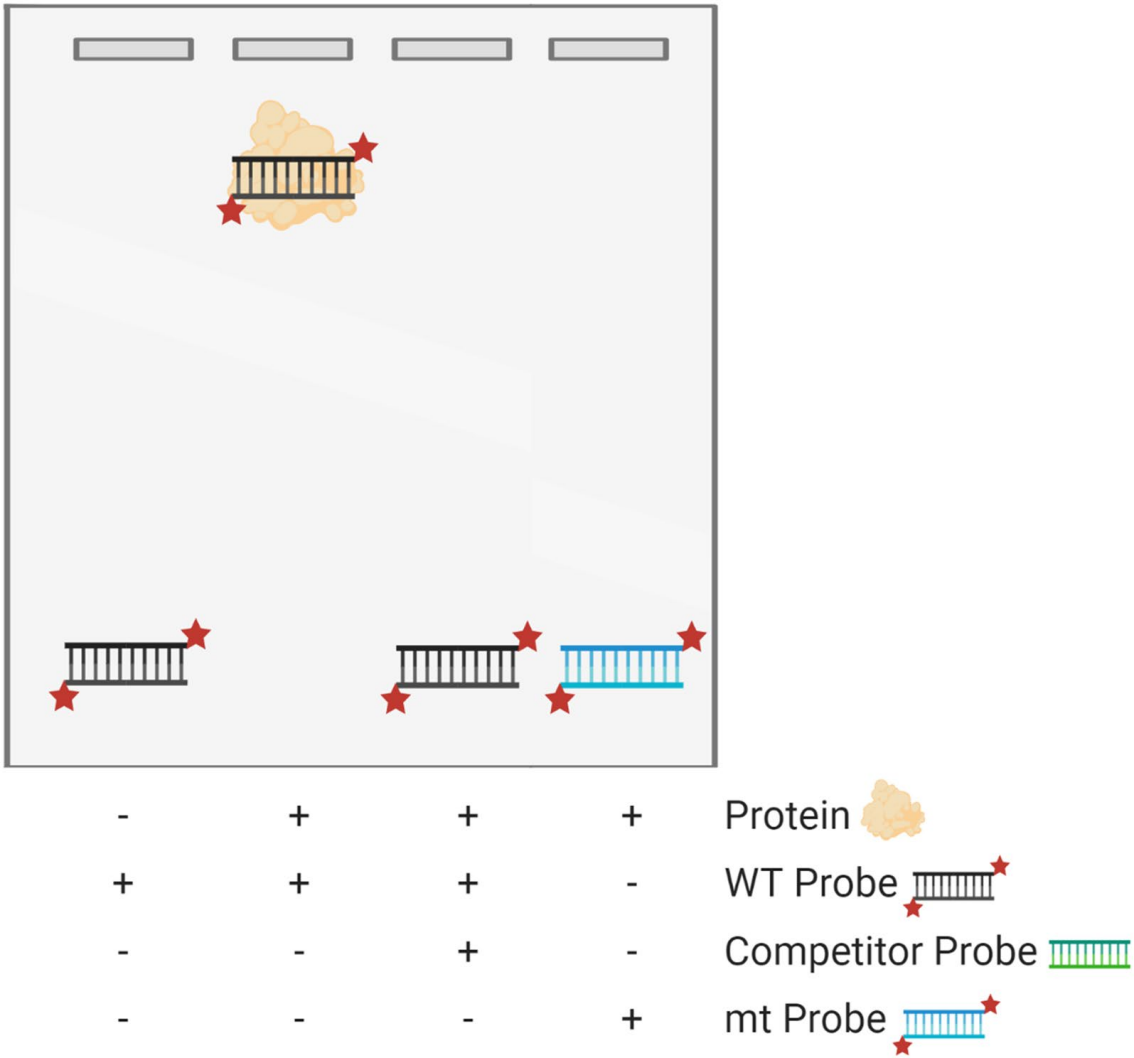
Schematic representation of the expected results of an EMSA visualised in an electrophoretic gel. While the first lane presents a band with the size of the segment being tested (WT probe), in the second lane, the introduction of the binding protein and formation of a nucleic acid–protein complex delays the run of this fragment. As for the third lane, the presence of an unlabelled competitor probe leads to the formation of a complex containing this competitor probe and the protein of interest and, consequently, the band that corresponds to the free labelled WT probe appears again. Finally, in the last lane, the replacement of the WT probe by a labelled mutated fragment (mt probe) may cause a similar result to the one of the fist lane, in case the mutation affects the complex formation. Created with BioRender.com

Usually, the technique starts by mixing the protein, present in a crude cell extract or purified, with the labelled nucleic acid and an appropriate buffer, under the right specific conditions and concentrations for the binding reaction to occur, and the final products are separated in a non-denaturing gel electrophoresis. To obtain purified protein, one can insert the coding sequence of the gene that codifies the protein of interest in an expression vector for bacteria or yeast transformation, induce the transcription and translation of this gene and extract and purify the protein using purification columns. As for the DNA fragments, these are usually labelled with radioisotopes, covalent or noncovalent fluorophores or biotin. The results are then observed by autoradiography, fluorescence imaging, chemiluminescent imaging and/or chromophore deposition (Fig. 2). It must be noted that certain parameters must be adjusted so that all this process succeeds, most of which were tested and resumed by Fried [20] and Hellman and Fried [18].

Such as filter binding assays [12, 16], the EMSA protocol can also include the use of a competing unlabelled nucleic acid, which is especially useful when dealing with crude extracts [3], where non-specific complexes are created by secondary binding activities. The incorporation of this additional reagent enables the discrimination of specific and non-specific unwanted bindings when the protein under study binds to the target nucleic acid with a higher affinity than it links to the competitor and when the other secondary binding activities do not differentiate between target and competitor sequences. Still, the competitor nucleic acid may also bind to the protein of interest, which will result in a decrease of the specific binding (Figs. 1, 2). In order to avoid this possibility, one should experiment several competitor concentrations with the purpose of improving the distinction between specific and non-specific interactions. Usually, poly d(A-T), poly d(I-C) and genomic DNAs are used as competitors for DNA–protein bindings [18], while tRNA is preferred when dealing with RNA–protein interactions [16].

Overall, EMSA is easy to execute, but powerful and sensitive, comprising a broad spectrum of binding conditions and enabling the use of small concentrations of protein and nucleic acid and small sample volumes, especially when the nucleic acid is radio-labelled. However, fluorescence, chemiluminescence and immunohistochemical detection can also be used in case a high sensitivity is not necessary. Concerning the advantages related to the nucleic acid used, this assay is able to test oligonucleotides with a large variety of sizes, which can go from short nucleic acids to molecules with thousands of nucleotides or base pairs, and structures, which can be single, double-stranded, triplex or quadruplex nucleic acids, or even small circular DNAs. As for the protein under study, proteins used in EMSA can have very distinct sizes and still efficiently provide mobility shifts. Using ESMA, one can even distinguish how proteins are spread across different interactions with various nucleic acid molecules in one solution, as well as the existence of complexes with different protein stoichiometry and/or binding site distribution [18].

Nevertheless, EMSA also presents some disadvantages. One of them is that, during the electrophoresis run, partial or total dissociation of the nucleic acid–protein complex can happen, or, on the contrary, many complexes acquire more stability in the gel than in a free solution. Either way, the results can be misleading, not reflecting the state of the interaction before the loading of the sample in the electrophoresis gel. To solve this issue, shorter electrophoresis times are suggested. Another drawback is that, in case a shift is observed in the mobility of a nucleic acid during the electrophoresis step, it is not necessarily due to the size of the proteins that are bound to it. Many other factors may be in the origin of the mobility shift, such as the conformation and structure of the complex. Moreover, this assay does not give information about the location of the sequences of the nucleic acid that are linked to the protein. These last two problems can be addressed using alternative techniques that complement or replace EMSA and that are referred in the next sections [18].

Overall, filter binding assay and EMSA are two important techniques that can help in identifying nucleic acid–protein interactions quite easily and rapidly. Advantages, disadvantages and applications of these two methods are resumed in Table 1. Other techniques may cooperate in order to obtain more information about this interaction. Some of them are examined in the following sections.

**Table 1:** Advantages and disadvantages of techniques that study DNA-binding proteins

Technique [References]	Technique		Applications	Case studies
	Pros	Cons		
Filter binding assay [12, 16–18]	Inexpensive and easy	The interaction may not withstand the filtration process Impossible to recover the resulting products	Identify nucleic acid–protein interactions	Prabu et al. [45]
EMSA [3, 18]	Fast and easy Powerful and sensitive Semi-quantitative		Identify nucleic acid–protein interactions Identify complexes in a cell/tissue extract	Liu et al. [46]
Cross-linking [3, 22]	UV cross-linking is not invasive and does not practically disturb the molecular structures Laser cross-linking is simple and fast and decreases the probability of damaging the molecular structures	Formaldehyde, glutaraldehyde and UV standard cross-linking methods are non-specific	Identifies the molecules that participate in a DNA–protein complex, even though some of them may not be directly in contact with the DNA	
EMSA combined with Western Blotting techniques [2, 18, 38]	Immunoblotting analysis combined with EMSA performs only one diffusion blotting Electrophoretic ‘supershift’ assay only uses one gel and does not need any diffusion blotting All variants are relatively fast and easy	Electrophoretic ‘supershift’ assay requires the purification of the antibody preparation Shift-Western Blotting involves two blotting membranes	Detect if a certain protein is present in a nucleic acid–protein complex (electrophoretic ‘supershift’ assay) and estimate its size (shift-Western Blotting and immunoblotting analysis combined with EMSA) Identify the nucleic acid–binding proteins that link to a certain nucleic acid and estimate their molecular weight (2D electrophoresis (EMSA + SDS-PAGE) and South-Western Blotting)	Wang et al. [47]
In vivo analysis: Y1H and PTA [42, 44]	EMSA and in vitro binding with a cell extract using mutated probes are quick and easy Y1H and PTA provide reliable results and are relatively direct and sensitive Y1H is quick	PTA takes a long time to be performed and involves several steps	Determine if a certain TF binds to a given sequence in vivo	Liu et al. [46]

#### Cross-linking

An EMSA technique drawback is the dependence of its results on several factors besides the protein size. This assay does not allow to identify nor reveal the molecular weights of the proteins present in the complex being studied. Obtaining this information is crucial especially when dealing with impurified or partially purified cell extracts, since they contain many nucleic acid–binding proteins, being that different proteins can bind to the nucleic acid of interest. Thus, other techniques need to be performed complementary to EMSA to clarify which protein is causing the electrophoretic mobility shift [18].

**Fig. 3:**
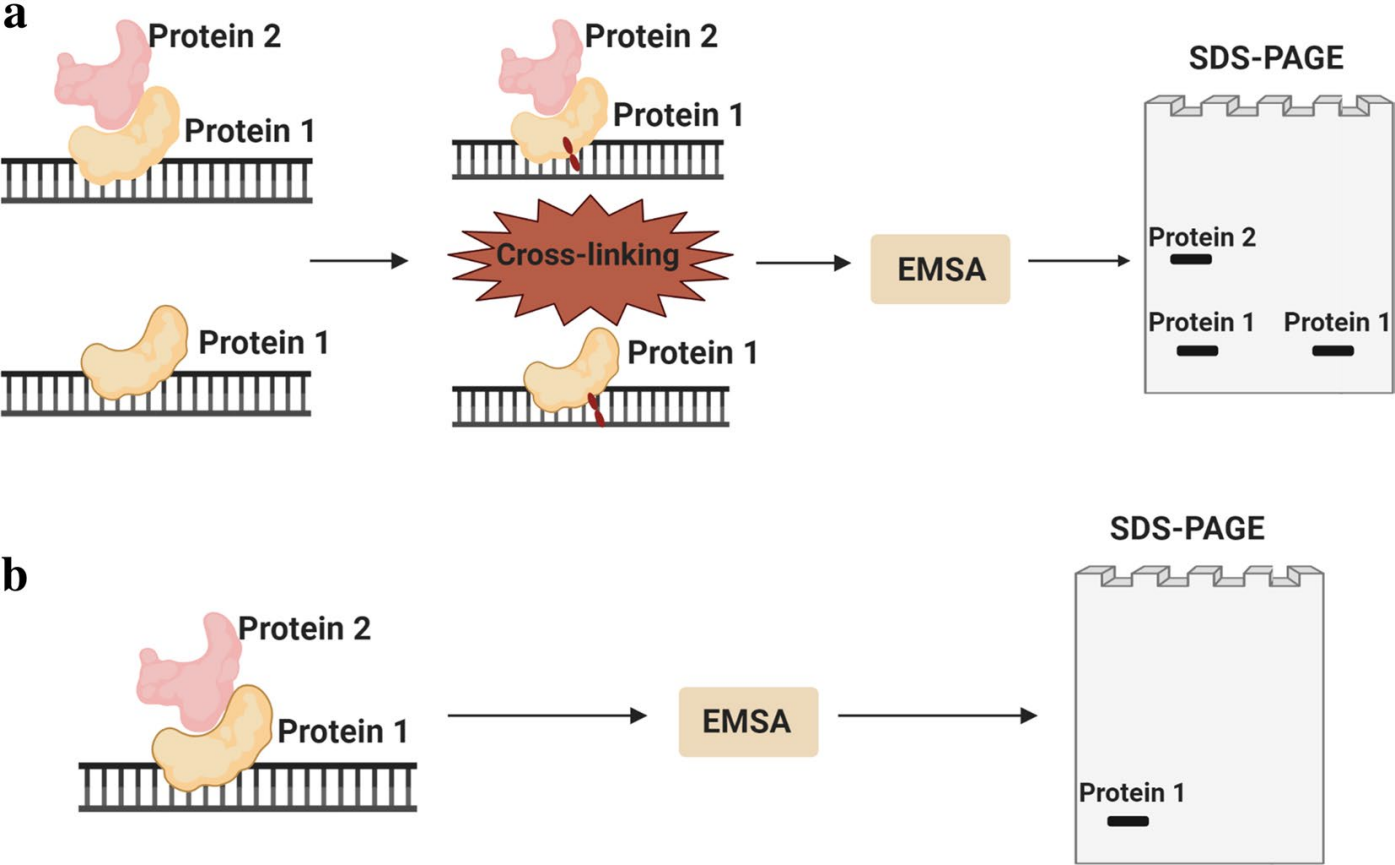
Comparison between the cross-linking procedure (**a**) and South-Western Blotting (**b**). In the cross-linking tehcnique (**a**) a nucleic acid is cross-linked to the respective binding proteins, ensuring that all proteins present in the complex are detected in a SDS-PAGE performed after an EMSA (that isolates the complex under study), independently of being in direct contact with the nucleic acid (Protein 1) or not (Protein 2). As for South-Western Blotting (**b**), after an EMSA, the complex is subjected to a SDS-PAGE without being exposed to a cross-linker and, consequently, only the proteins in direct contact with the nucleic acid will be detected. Created with BioRender.com

In the seventies, quaternary structures related to interactions between proteins were analysed using cross-linking techniques [21]. Covalent cross-links were produced between proteins belonging to the same complex, so that they could be extracted and studied in subsequent steps. Other approaches also developed, enabled the generation of cross-links between DNA and proteins from the same complex [22]. Generally, each distinct procedure starts by forming or isolating the DNA–protein complexes, excluding free probes and nonspecific complexes. A cross-linker agent is then applied and the specific complex is removed and precipitated, being finally analysed [3] (Fig. 3a). In some of the following approaches, the cross-linker may be applied prior to the formation of the complex to increase the affinity of the DNA to bind to proteins.

There are different cross-linker agents and strategies, which can be grouped into chemical and photo-cross-linking. Chemical cross-linking techniques often use formaldehyde and glutaraldehyde as the cross-linker agents. These two agents are usually employed in the study of nucleoproteins, such as histones [22]. Brutlag et al. [23] found that the presence of DNA in formaldehyde treatment of nucleohistones generates DNA–protein complexes, due to the methylene bridges formed between adjacent amino groups. It was also reported by the same researchers that the increase of the formaldehyde concentration results in a higher amount of bounded protein. However, they concluded that high concentrations of formaldehyde causes secondary alterations in the histones structure or composition, which can be a disadvantage of this cross-linker agent [23]. As for glutaraldehyde, while Chalkley and Hunter [24] stated that, unlike formaldehyde, glutaraldehyde creates bonds primarily between histones, without excluding a few DNA-histone links, Kuykendall and Bogdanffy [25] reported that glutaraldehyde is a very potent cross-linker, almost as effective as formaldehyde, most probably because of its difunctional nature. Chemical cross-linking can also be applied when studying virus structures, as it was performed by Chattoraj and Inman [26], that used formaldehyde as a cross-linker agent when trying to unravel the arrangement of DNA in bacteriophage heads, and Thomas et al. [27], who employed 1-ethyl-3(3-dimethyl-aminopropyl) carbodiimide with a similar purpose.

Photo-cross-linking was firstly performed by Smith [28], that irradiated DNA mixed with protein with a UV light of 254 nm, near the absorbance maximum for DNA and some amino acid side chains, using a mineral lamp. This causes cross-link of the DNA to several distinct amino acid side chains, being a non-specific method. Among other works, this method was applied in studying cross-linking between histones and DNA and virus replication [29, 30]. This technique’s advantage lies in the fact that this procedure is not invasive and does not practically disturb the structures of the molecules under study [22]. Nevertheless, the method was improved. Two decades after the first cross-linking experiment using UV light, Harrison et al. [31] inflicted a very short UV pulse from a laser on a complex formed by *E*. *coli* RNA polymerase and T7 DNA, inducing the cross-link between these two molecules. Apart from being a simple and fast method, this upgrade to the UV cross-linking technique decreases even more the chance of damaging the structure of the molecules being analysed, since it uses short and powerful pulses. Besides, this approach can also be applied in in vivo studies, without affecting the metabolism of a cell, as it happens with conventional UV cross-linking. It also enables the immediate cross-link of a complex involving several proteins. However, this procedure may cause more chain scission in DNA than the standard UV cross-linking method referred before [31].

Overall, cross-linking techniques have the advantage of identifying the molecules that participate in a DNA–protein complex, even though some of them may not be directly in contact with the DNA. Nevertheless, when analysing the molecular weight of the proteins present in the complex through a sodium dodecyl sulphate–polyacrylamide gel electrophoresis (SDS–PAGE) (Fig. 3a), DNase I might be necessary in order to remove the DNA attached to the proteins, risking a decrease of the signal, since the presence of DNA may lead to an irregular run, giving an incorrect estimation of the molecular weight [3]. Advantages, disadvantages and applications of some cross-link variants are resumed in Table 1.

#### EMSA combined with western blotting techniques

Western Blotting was initially developed by Towbin et al. [32] enabling the identification of proteins using specific antibodies, after performing an electrophoresis. It can be used to detect specific proteins present in a cell extract. Several techniques were developed that combine EMSA with Western Blotting, making it possible to detect a specific DNA–protein interaction, as well as the size of the protein, within a cell extract.

Kristie and Roizman [33] created an approach called electrophoretic ‘supershift’ assay, which consists in adding to the nucleic acid–protein mixture an antibody against a candidate protein. In case the protein causing the mobility shift of the nucleic acid is the one of interest, the presence of the antibody will cause a secondary mobility shift, or the blocking of the complex formation. As for the moment when the antibody is added, the results obtained with this method will be different if the antibody is included before or after the formation of the nucleic acid–protein complex, mainly if the target protein contains epitopes in the DNA-binding surface. Moreover, the antibody preparation needs to be as pure as possible, since contaminants may interfere in the stability or mobility of the nucleic acid–protein complexes. Alternatively, a control antibody reaction can also be prepared to detect possible interferences of the antibody preparation [18, 34].

Singh et al. [35] also created a technique fusing EMSA and Western Blotting, the South-Western Blotting, according to which the complex identification uses radioactively labelled oligonucleotides. After separating the cell proteins by SDS-PAGE and transferring to nitrocellulose filters, these are either incubated with an oligonucleotide corresponding to the nucleic acid of interest, or with a point-mutated variation of the same oligonucleotide that does not form the specific complex, serving as control. An EMSA is performed previously to verify the adequacy of the control oligonucleotide designed. The filters are then washed and exposed for autoradiography (Fig. 3b). The differences between the results obtained with each oligonucleotide represent the specific nucleic acid–protein complexes. If more than one specific band is observed, it means that the nucleic acid sequence under study is recognised by a family of proteins. Although providing precise information about the molecular weight of a nucleic acid–binding protein, this procedure only succeeds if the protein is in direct contact with the nucleic-acid and if the nucleic acid binding does not involve an heteromeric protein complex [3].

Demczuk et al. [36] performed a method involving simultaneous immunoblotting analysis with EMSA, known as ‘shift-Western Blotting’, according to which a nitrocellulose filter and an anion-exchange membrane are piled following native gel electrophoresis to identify the nucleic acid–protein complex by autoradiography of the DNA blot, present in the anion-exchange membrane, and by immunoblotting of the protein blot, obtained in the nitrocellulose filter. However, Granger-Schnarr et al. [37] and Chen and Chang [38] created a combined analysis that only performs one simple diffusion blotting into a polyvinylidene difluoride (PVDF) membrane after electrophoresis, being the gel directly analysed by autoradiography and the PVDF incubated with a specific antibody and analysed by immunoblotting. This last procedure is especially powerful, since it enables the identification of each component of a multiple DNA-TF complex, replicating the immunoblotting analyses using antibodies specific for each TF. Finally, in 2006, Stead et al. [39] implemented a two-dimensional gel electrophoresis, that combined EMSA, SDS-PAGE and mass spectrometry in the same gel. After performing an EMSA in a tube gel, this tube is placed above an SDS gel in order to separate the proteins present in the specific complexes obtained in the EMSA according to their molecular weight, which are then identified performing a peptide mass fingerprinting. This method enables the detection of nucleic acid–protein complexes in a cell extract within 4 days, significantly decreasing the need for protein purification and facilitating the identification of all the proteins present in that extract that interact with the nucleic acid being studied. Advantages, disadvantages and applications of some techniques that combine EMSA with Western Blotting are resumed in Table 1.

#### In vivo analysis: Y1H and PTA

In order to relate binding sites with the TF binding in vivo, systems like the yeast one-hybrid (Y1H) were created. This system, developed by Li and Herskowitz [40], implicates a vector expressing the TF under study fused to a yeast transcription activation domain, usually GAL4, and a construct containing the gene of interest, or a region of its promoter, more specifically, its *cis* elements, upstream to the GAL4 promoter and a reporter gene, usually the *lacZ* gene. These two elements are inserted into a yeast cell and, in case the TF does bind to the gene of interest, the activation domain binds to the promoter and leads to the expression of the reporter gene, being this activation independent of the TF action, either it is an activator or a repressor. Unlike in vitro techniques, this technique employs a system where a cell is involved, providing much more reliable results since it recreates an environment similar to the one where the complex under study is formed [41]. It can be concluded that Y1H is a relatively direct, quick and sensitive method to determine if a certain TF binds to a given sequence in vivo [42].

**Fig. 4:**
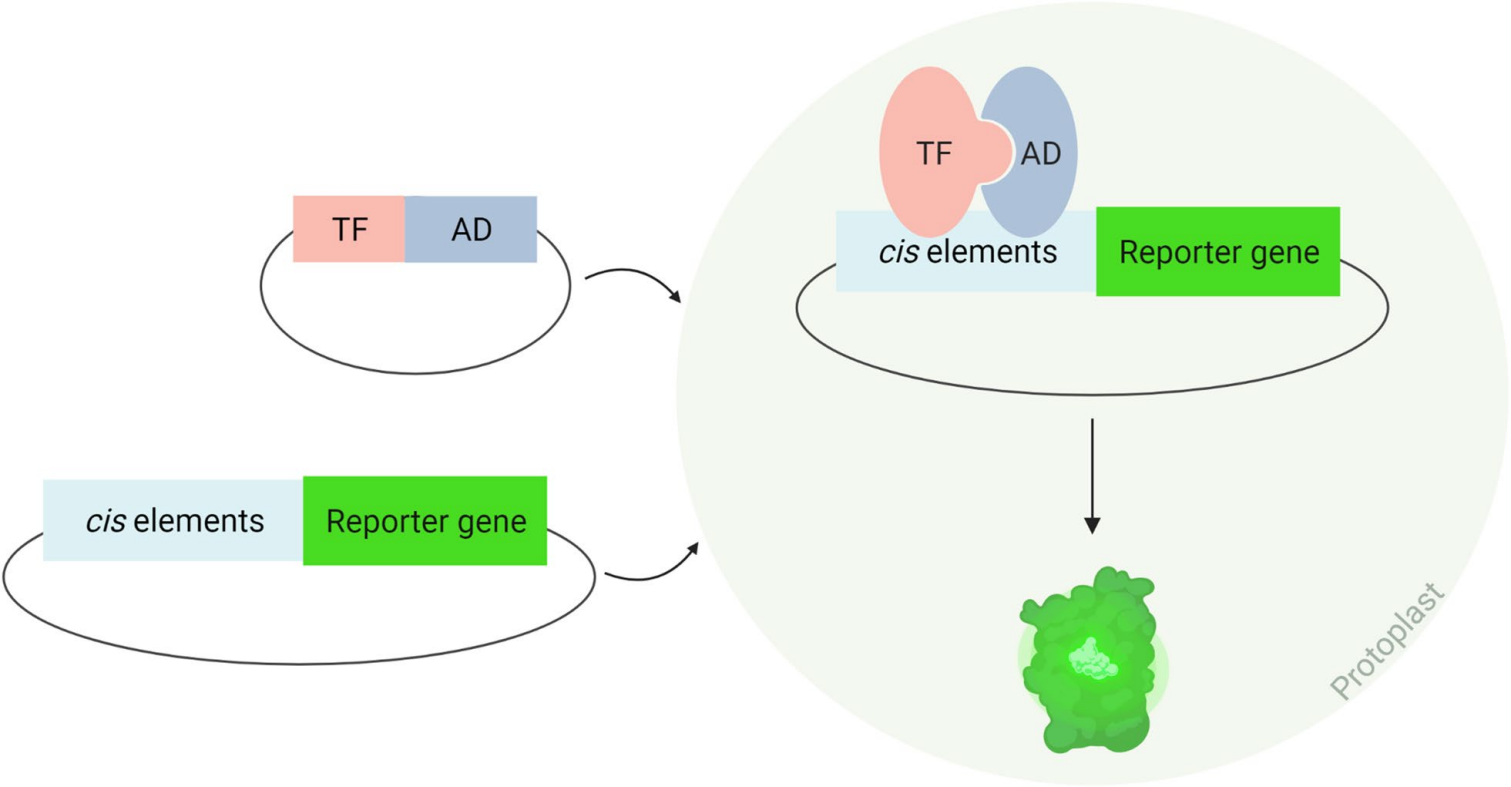
Overall schematic representation of the PTA procedure. In this technique, two vectors are inserted into a protoplast: one containing the gene of the TF being tested (TF), right next to the gene of an activation domain (AD); a second one that embraces the sequence of the *cis* elements of the target gene upstream of a reporter gene. Inside the protoplast, after the expression of the TF gene, in case the TF binds to the target gene *cis* elements, the reporter gene expression is activated by the activation domain fused to the TF. Created with BioRender.com

Still, a yeast cell is quite different from other cells, such as a plant cell. This method does not totally recreate the environment of a plant cell, disregarding some factors that might interfere and determine the DNA–protein interaction being studied. Therefore, in this case, an *in* *planta* was developed. The creation of a Transient Expression in Arabidopsis Mesophyll Protoplast (TEAMP) method by Yoo et al. [43], a protoplast transactivation assay (PTA), enabled to perform an experiment similar to Y1H, but using plant cells, more specifically protoplasts, eliminating the risk of drawing false conclusions from techniques that use different organisms, like mentioned above. Ueda et al. [44] used this protocol to study the interaction between the *Wuschel* *related* *homeobox* *8* (*WOX8*) gene and the WRK2 TF. After identifying the *WOX8* *cis* elements, the group followed the TEAMP protocol and transformed Arabidopsis mesophyll protoplasts, previously transformed with another vector containing the *WRKY2* gene linked to the GAL4 activation domain, with the constructs containing the *cis* elements linked to the GAL4 promoter and a reporter gene. As these protoplasts exhibited reporter gene expression, the group suggested that WRKY2 does bind to the *WOX8* gene, which, in this experience, led to the expression of the reporter gene (Fig. 4). Despite being a very complex procedure that involves numerous steps, PTA is a reliable *in* *planta* technique when trying to decipher gene expression networks and study gene expression direct regulation. Advantages, disadvantages and applications of the techniques referred are resumed in Table 1.

### DNA-binding site localisation

#### Footprinting

A method usually performed in order to discover the precise sequence of the nucleic acid that binds to a protein is the footprinting. There are several footprinting techniques, each one with a different way of testing if a modification in a particular segment of the nucleic acid interferes or is blocked by protein binding, enabling to identify the location of the binding sequence. All the footprinting variations have in common the ^32^P end-labelling of the nucleic acid strands being tested; the modification and binding of each nucleic acid strand in parallel reactions; the sequencing of each fragment to identify the modification sites; and, if possible, the quantitative analysis of the “free” and “bound” nucleic acid fractions after sequencing. Footprinting techniques can be divided into “protective” and “interference” methods, being each one the reverse of the other. While in the first the protein binds to the nucleic acid before and “protecting” it from modification, in the latter the nucleic acid is altered first and then tested for protein binding [3].

**Fig. 5:**
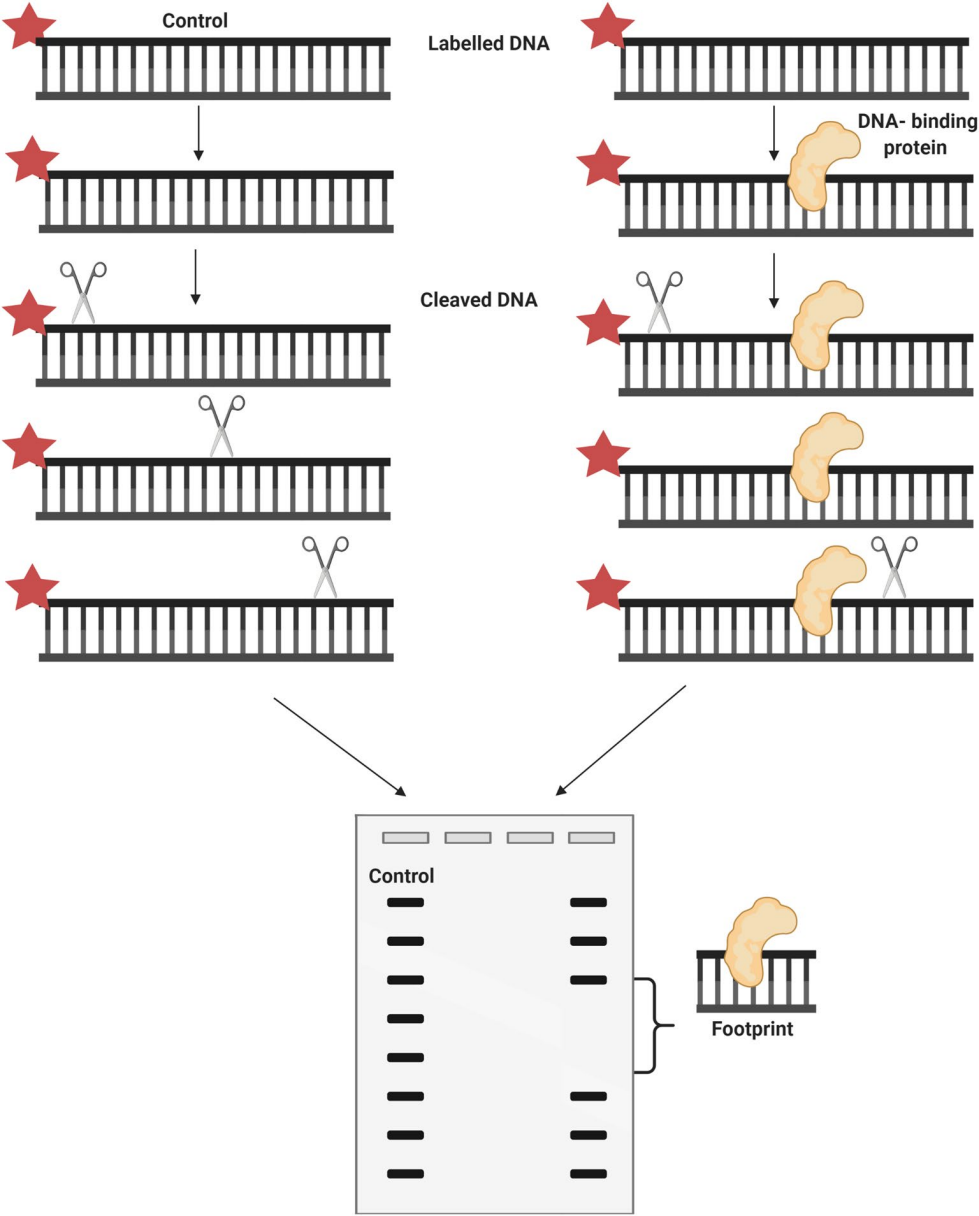
Representation of the “protective” footprinting technique. This method starts by mixing the nucleic acid of interest with the binding protein under study (on the right). Then, an enzymatic or chemical reagent cleaves the nucleic acid, except for the region protected by the protein, in case a complex is formed between the nucleic acid and the protein. The cleaved nucleic acid is submitted to a denaturing electrophoresis in order to identify the protected segment, represented by a gap in the bands of the restriction products, the footprint, when compared to a lane containing the free DNA cleavage products (control) (on the left). Created with BioRender.com

“Protective” footprinting methods, in turn, can be divided into enzymatic or chemical. In both approaches, the nucleic acid of interest is mixed with the protein under study, whether it is present in a crude extract or in a pure sample. Then, the enzymatic or chemical reagent partially cleave the nucleic acid, except for the region protected by the protein, in case it binds to the nucleic acid. Finally, the cleaved nucleic acid is submitted to a denaturing electrophoresis, together with a sequencing ladder, and to autoradiography in order to identify the protected segment, represented by a lacuna in the continuous bands of the restriction products, the footprint, when compared to a track containing the free DNA cleavage products [3] (Fig. 5).

“Protective” footprinting results usually present a background that corresponds to free DNA that was not linked to a protein. This problem can be overcome by adding large amounts of protein, which is not always easy to obtain, especially when dealing with purified samples, and can lead to non-specific binding. Another way to eliminate the background is to perform an EMSA, in order to separate free- and bound-DNA fractions, prior to the footprinting reaction [3]. Papavassiliou and Heumann [48] developed a new method according to which the footprinting reaction is executed in the nondenaturing gel of the EMSA. Thus, the complex is maintained in the gel while being exposed to the footprinting reagents. Moreover, multiple complexes can be separately obtained and subjected to the footprinting agent, enabling to study different binding sites and complexes in the same nucleic acid fragment. Another disadvantage of these in vitro footprinting methods is the disregard for factors existing in cells that may interfere in the complex formation [3]. Nonetheless, Church and Gilbert [49] developed an in vivo footprinting method, starting by treating cells with DMS, since it can penetrate plasma and nuclear membranes. After isolating the genomic DNA of these cells, the altered guanine residues are restricted by piperidine.

Relatively to “interference” footprinting methods, these techniques consist in inflicting modifications on DNA bases and identifying the alterations that inhibit or diminish protein binding. These approaches are more direct and have a higher resolution than the previous ones, since they investigate the impact of each nucleotide on the binding affinity, modifying only one nucleotide per DNA fragment [3].

Overall, footprinting techniques present a low throughput [50] and need optimisation of binding by the protein under study and of the modification reaction of the nucleic acid, being harder to perform than EMSA or filter binding assays. Furthermore, as many nucleic acid fragments are radiolabelled in footprinting methods, the detection of the binding is less sensitive than using EMSA. In addition, given that some proteins bind to nucleic acids in a non-specific way, the results obtained with footprinting procedures may not be so distinct as the ones achieved by EMSA and filter binding assays. Nevertheless, footprinting is the method mostly chosen to identify the sequences of the binding sites. This method can also be applied to study the interactions of a long nucleic acid with several binding proteins, given that big sequences can be resolved on a conventional sequencing gel. Moreover, contrary to EMSA, a footprint signal can be visualised in binding equilibrium conditions [18]. Advantages, disadvantages and applications of the footprinting techniques are resumed in Table 2.

**Table 2:** Advantages and disadvantages of techniques that study DNA-binding sites

Technique [References]	Technique		Applications	Case studies
	Pros	Cons		
Footprinting [3, 18, 50]	In vivo methods consider factors existing in cells and enable the analysis of the transcription of particular alleles useful to study the interactions of a long nucleic acid with several binding proteins Signal is observed in binding equilibrium conditions	Low throughput “Protective” footprinting usually presents a background In vitro methods disregard factors existing in cells Need improvement of binding by the protein under study and the nucleic acid modification reaction The detection is not too sensitive The results obtained may not be so distinct	Discover the precise sequence of the nucleic acid that binds to a protein “Interference” footprinting: investigate the impact of each nucleotide on the binding affinity	Manosas et al. [55]
Base analogues [3]	Provides accurate results Access the relative affinity of a protein for DNA fragments that lack particular groups on a base	Expensive and long procedure	Identify the contribution of each base to the DNA-binding affinity of a protein Understand the contribution of crucial interactions to the binding	
Binding-site selection [3, 42]	Provides accurate results Does not depend on cloning Does not require the analysis of phenotypes SELEX-SAGE provides binding-site models with a higher accuracy High-throughput SELEX provides better fits to the selected site distributions	Becomes more difficult when dealing with TFs that bind to several genes	Rapid selection of the oligonucleotides that have appropriate binding affinity to a molecular target from a library of randomly generated oligonucleotides	Prabu et al. [45]

#### Base analogues

An expensive and long procedure that can be performed to identify the contribution of each base to the DNA-binding affinity of a protein involves the integration of modified bases at particular sites in the course of the synthesis of both strands that create the DNA duplex [51]. This technique involves assembly and an EMSA test for each specific DNA duplex designed for every alteration being analysed. However, this method provides more accurate results than the footprinting procedures. It enables to access the relative affinity of a protein for DNA fragments that lack particular groups on a base, allowing to understand the contribution of crucial interactions to the binding. This procedure is quite useful to determine the precise bases interfering in the DNA–protein interaction, especially if complementing previous experiments that have pointed towards a specific binding site [3]. The advantages, disadvantages and applications of this method are present in Table 2.

#### Binding-site selection

**Fig. 6:**
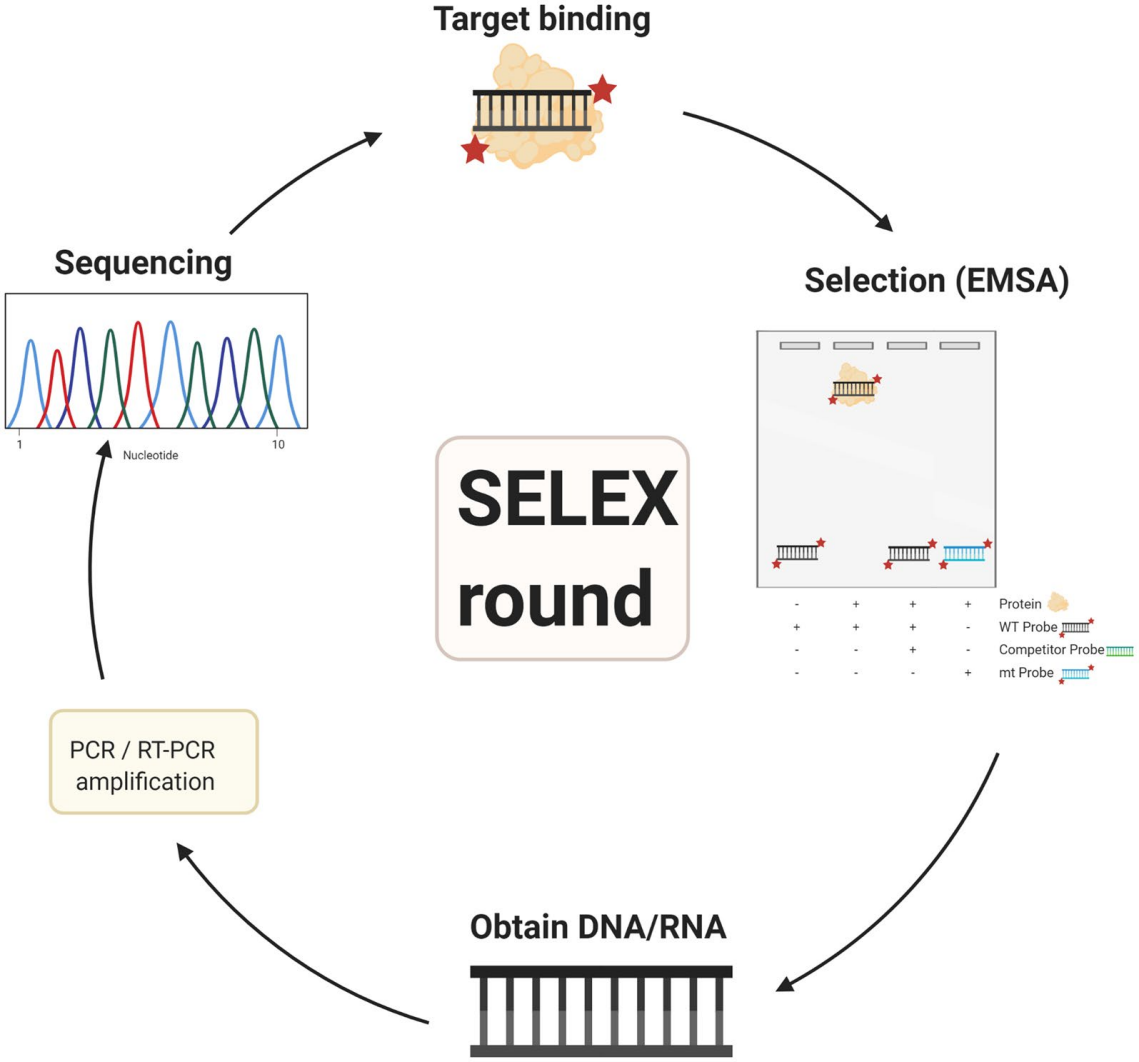
Scheme of a SELEX round. After creating a library of oligonucleotides and incubating them with the target protein, the bound and free fractions are separated, usually through an EMSA, and the bound oligonucleotides are amplified by PCR, regarding DNA, or by RT-PCR succeeded by in vitro transcription, concerning RNA. This procedure is called a SELEX round and is repeated several times, being some oligonucleotides chosen in the last round and sequenced. Created with BioRender.com

Another radical technique consists in synthesising oligonucleotides that incorporate a random nucleotide mixture at specific positions in a certain sequence. The bases located at determined sites in sequences that bind to the protein under study interaction, usually checked by an EMSA, are established as the preferred bases [3]. In the 90’s, new methods were created, that gave a great contribution to the techniques that select binding-sites from random oligonucleotide mixtures. Tuerk and Gold [52] developed the systematic evolution of ligands by exponential enrichment (SELEX) procedure, an in vitro technique that rapidly accesses the oligonucleotides that have an adequate binding affinity to a certain molecular target from a library of random generated oligonucleotides. Basically, this method starts by creating a library of oligonucleotides, followed by incubation with the target protein. Subsequently, the bound and free fractions are separated and the bound oligonucleotides are amplified by PCR, regarding DNA, or by RT-PCR succeeded by in vitro transcription, concerning RNA. This three-strep procedure, target binding, selection and amplification, is called a SELEX round and is repeated numerous times, being some oligonucleotides selected in the last round, called aptamers, sequenced (Fig. 6). In previous techniques, random nucleotides were cloned into plasmids that were then used in transformation procedures and individual transformants were selected according to their phenotypes. Thus, the SELEX method presents several advantages over the procedures performed until its creation. Firstly, it does not depend on cloning to select the oligonucleotides. Furthermore, it does not require the analysis of phenotypes, which may depend on various processes and other potential in vivo variables. This quite simple procedure identifies the ideal binding sequences for a given protein only requiring a relatively pure target protein sample, a method like EMSA to separate the bound and free fractions and a PCR to amplify the selected oligonucleotides. Moreover, the oligonucleotides used can be single or double-stranded DNA or RNA [52]. However, this technique also presents a drawback: several DNA-TF interactions have a low level of specificity and sensitivity. Prediction of the binding sites becomes more difficult when dealing with TFs that bind to several genes, presenting binding motifs with low information content [53]. Nevertheless, Roulet et al. [54] developed a high-throughput genomic method that fused SELEX with serial analysis of gene expression (SAGE) and an automated procedure to extract quality-controlled sequences, providing binding-site models with a higher accuracy and identifying additional regulatory sequences in genomic DNA. The advantages, disadvantages and applications of each one of these approaches are described in Table 2.

### DNA–protein binding quantification

#### Filter binding assay and EMSA

Apart from identifying nucleic acid–protein complexes and characterising these proteins, the filter binding assay and EMSA can also be useful to distinguish the complexes formed according to the equilibrium constants of each binding reaction, enabling to compare relative binding affinities and discriminate the interaction between a given protein with more than one nucleic acid sequence. To do so, it is essential to use a purified protein and know the respective concentration. Then, the affinity of a protein to a certain nucleic acid can be quantified performing a titration of the protein under study with a known concentration into a permanent concentration of the nucleic acid of interest [3].

Usually, the Michaelis–Menten analysis can be applied to examine these titrations, considering the protein added as the substrate, since the nucleic acid concentration is very low. This demands a high protein concentration to obtain a significant interaction, being the free and total protein concentrations quite similar. Thus, the equilibrium constant can be estimated as the concentration of protein necessary to retain, as for the filter binding assay [15], or shift the mobility, regarding the EMSA [17], of 50% of the nucleic acid. Furthermore, the increase in the amount of protein is accompanied by an increase in the amount of complex formed, until reaching a plateau, that corresponds to a saturation level, quite like the Michaelis–Menten curve [56]. Nevertheless, this analysis assumes a 1:1 stoichiometry and that all the protein is active [3].

Analysing DNA–protein interactions by intrinsic fluorescence, Carpenter et al. [57] developed a model according to which the use of high nucleic acid concentrations leads to a binding linear to the protein concentration (*P*) until all the DNA is bound. At this point, the ratio between the protein and DNA concentrations gives the stoichiometry. If it is higher than 1, it can mean that more than one protein link to the DNA, or that part of the protein is not active. Moreover, taking into consideration that, at the same moment, the concentration of the complex formed (*PD*) is equal to the DNA concentration (*D*) and that the equilibrium constant (*K*) is calculated using the formula:

$$K=\frac{PD}{P\times D},$$

at this point K is equal to $\frac{1}{{\text{P}}}$ [3]. However, in order to determine a high affinity, the protein concentration needs to be very low, which is difficult to measure. This way, in these cases, one can perform serial dilutions and calculate *P* by the formula:

$$P={P}_{total}-PD,$$

being the total concentration of protein (*P*_*total*_) calculated for each dilution and the *PD* directly measured [58].

Following the same model, for intermediate DNA concentrations, when the stoichiometric point is reached, only a part of the DNA (*f*) is linked to the protein (*P*). Here the equilibrium constant (*K*) is easily and accurately calculated by the formula:

$$K=P\times \frac{{(1-f)}^{2}}{f}.$$

However, unlike the previous case, here the DNA concentration needs to be acquired [3, 57].

Finally, competition assays can also be performed and equilibrium constants can be compared. In a first approach, the relative binding constant (*K*_*r*_) can be calculated using the formula:${K}_{r}=\frac{K}{{K}_{c}},$being *K*_*c*_ the equilibrium constant of the reaction where the competing nucleic acid binds to the protein. This formula also represents the equilibrium constant of the overall reaction (*K*_*T*_). Taylor et al. [59] calculated the same overall equilibrium constant using the concentration (*C*) of the competitor sequence needed to dissociate half of the specific complex and the formula.

$${K}_{T}=2\times \frac{C}{D}-1.$$

Nevertheless, automated systems needed to be created. Initially developed by Gassmann et al. [60] and adapted for the study of DNA–protein interaction by Xian et al. [61], capillary electrophoresis–laser-induced fluorescence (CE-LIF) consists in submitting a DNA–protein complex to a capillary EMSA, using a laser-induced fluorescence detection system. It allows instant on-column visualisation, automated operation and computerised data analysis, enabling DNA–protein complex and DNA quantification and, consequently, stoichiometry determination. Moreover, it has small sample requirements, is highly sensitive and presents rapid analysis times [62]. Furthermore, combining CE with laser-induced fluorescence polarisation enables simultaneous measurements of electrophoretic mobility and fluorescence anisotropy ("Binding-site selection" section) [63]. Table 3 presents the advantages, disadvantages and applications of each one of these methods.

**Table 3:** Advantages and disadvantages of techniques that describe DNA-binding reactions

Technique [References]	Technique		Applications	Case studies
	Pros	Cons		
Filter binding assay and EMSA [3, 20, 62]	The Michaelis–Menten analysis can be applied use of intermediate DNA concentrations enables to determine directly and accurately the equilibrium constant The use of high DNA concentrations enables to determine the stoichiometry CE-LIF enables instant on-column visualisation, automated operation and computerised data analysis CE-LIF has small sample requirements, is highly sensitive and presents rapid analysis times	Need to use purified protein and quantify its concentration The use of low DNA concentrations assumes 1:1 stoichiometry Need to perform serial dilutions to determine high affinities	Compare binding affinities CE-LIF enables DNA–protein complex and DNA quantification and stoichiometry determination CE-LIF enables simultaneous measurements of electrophoretic mobility and fluorescence anisotropy	Prabu et al. [45]
SPR [42]	Very sensitive, fast and easy Real-time assay More adequate than EMSA when comparing wild-type and mutant proteins	If more than one protein bind cooperatively, the results can be misleading	Measure binding affinities and kinetics directly and simultaneously	Song et al. [74]

#### Surface plasmon resonance

In the beginning of the twentieth century, Wood [64] observed a dark and light bands pattern in the light reflected from a metal-backed diffraction grating exposed to polarised light. This phenomenon was explained later by Fano [65] as being related to surface waves, surface plasmon, supported by the grating. More than two decades after this theoretical analysis, Otto [66] demonstrated optical excitation of surface plasmons through attenuated total reflection. Thus, two ways of optically excite surface plasma waves are: attenuated total reflection in prism coupler-based structures and diffraction at gratings. Liedberg et al. [67] were pioneers in applying the first approach in sensing chemical substances, introducing the surface plasmon resonance (SPR) sensing technique, while Cullen et al. [68] were the first ones to make use of the second method for the same purpose. In the beginning of the 90’s, the field of bioanalysis with surface plasmon resonance had a huge development with the creation of SPR-biosensors machines, which apply the attenuated total reflection approach and include a dextran layer on the surface of a thin gold film, a laser beam and a diode array for detection [69]. Kinetic analysis of binding reactions [70] and DNA–protein interactions studies [71] that used these biosensors followed.

**Fig. 7:**
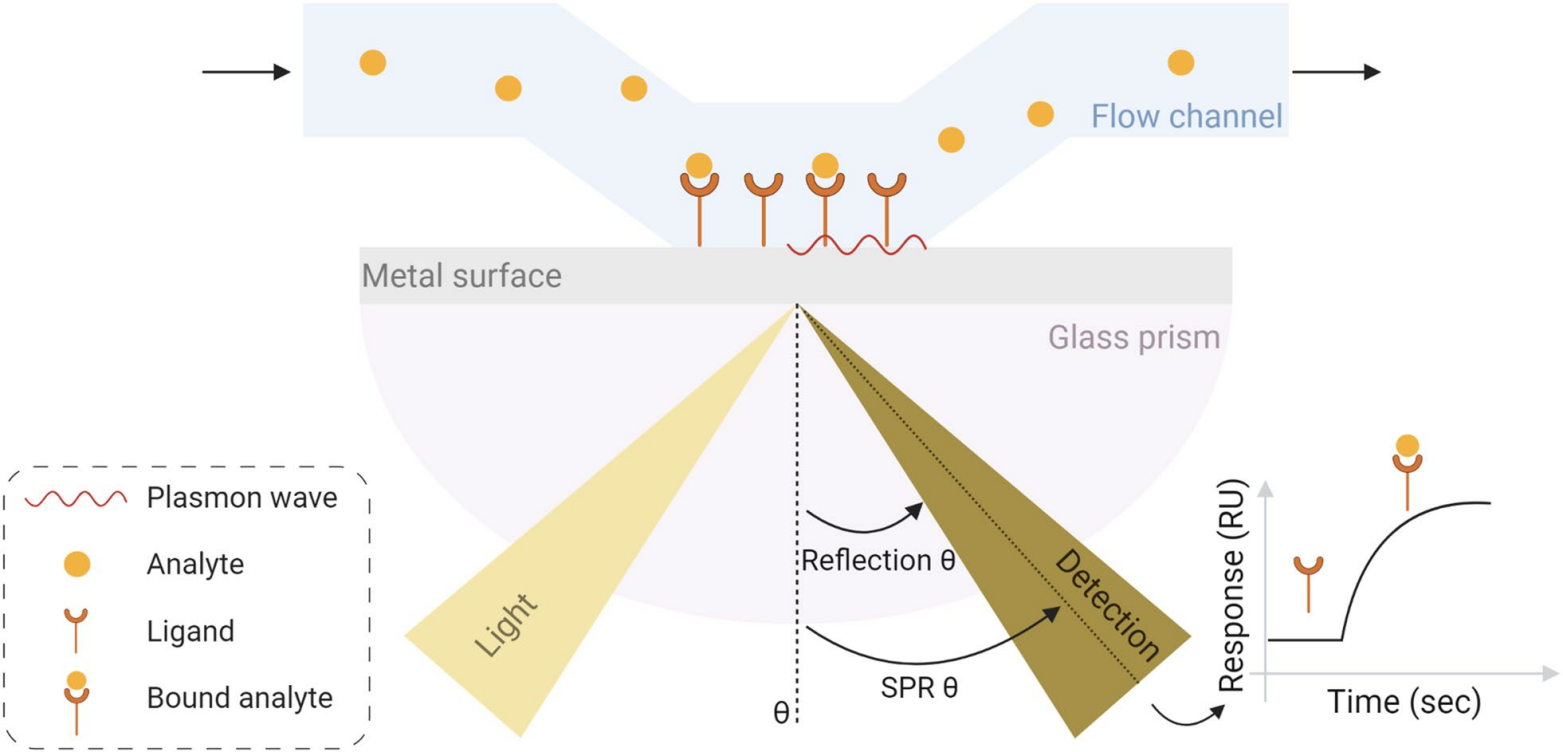
Illustration of the SPR method. It starts by arresting one of the binding partners (ligand) to the surface of a metal surface, followed by injecting the other binding partner (analyte) through a flow channel. A light source emits a single wavelenght light which is directed through a glass prism to the back of the metal surface, being then reflected to a detector with a given angle (reflection θ) and creating a plasmon wave. The binding between the analyte and the ligand causes a shift in the angle at which the light is absorbed (SPR θ). A real-time interaction curve records these oscillations (in resonance units [RU]) as a function of time. Created with BioRender.com

Basically, the SPR protocol for measuring biomolecular interactions starts by arresting one of the binding partners to the surface of a chip, followed by injecting the other binding partner. A real-time interaction curve is then recorded, measuring the increase in mass due to the binding as a function of time (Fig. 7). Flowing buffer over the chip leads to the dissociation of both partners and the signal decreases. This way, the kinetics of the interaction can be studied and the association and dissociation rate constants can be accessed [3].

Although, if more than one protein binds cooperatively, the results obtained with the SPR technique can be misleading and need a more complex analysis [3], SPR presents several advantages. It is a very sensitive method that directly measures binding affinities and kinetics simultaneously. Furthermore, this technique does not require labelling of the binding partners and is a real-time assay. Relatively to EMSA, SPR is faster, easier and more adequate when comparing wild-type and mutant proteins [42]. Even though, the immobilisation of the ligand in the SPR assay may affect its activity [72], Khan et al. [73] developed a new SPR approach where the protein binds to the chip at physiological conditions, which, relatively to standard immobilisation techniques, is fast, efficient and reversible. The advantages, disadvantages and applications of SPR are present in Table 3.

### Genome-wide techniques: ChIP and respective variants

DNA fragments characteristics and sequences alone cannot be used to predict the genomic locations of bound proteins in a particular cell type. Thus, functional genome-wide approaches needed to be developed [75].

**Fig. 8:**
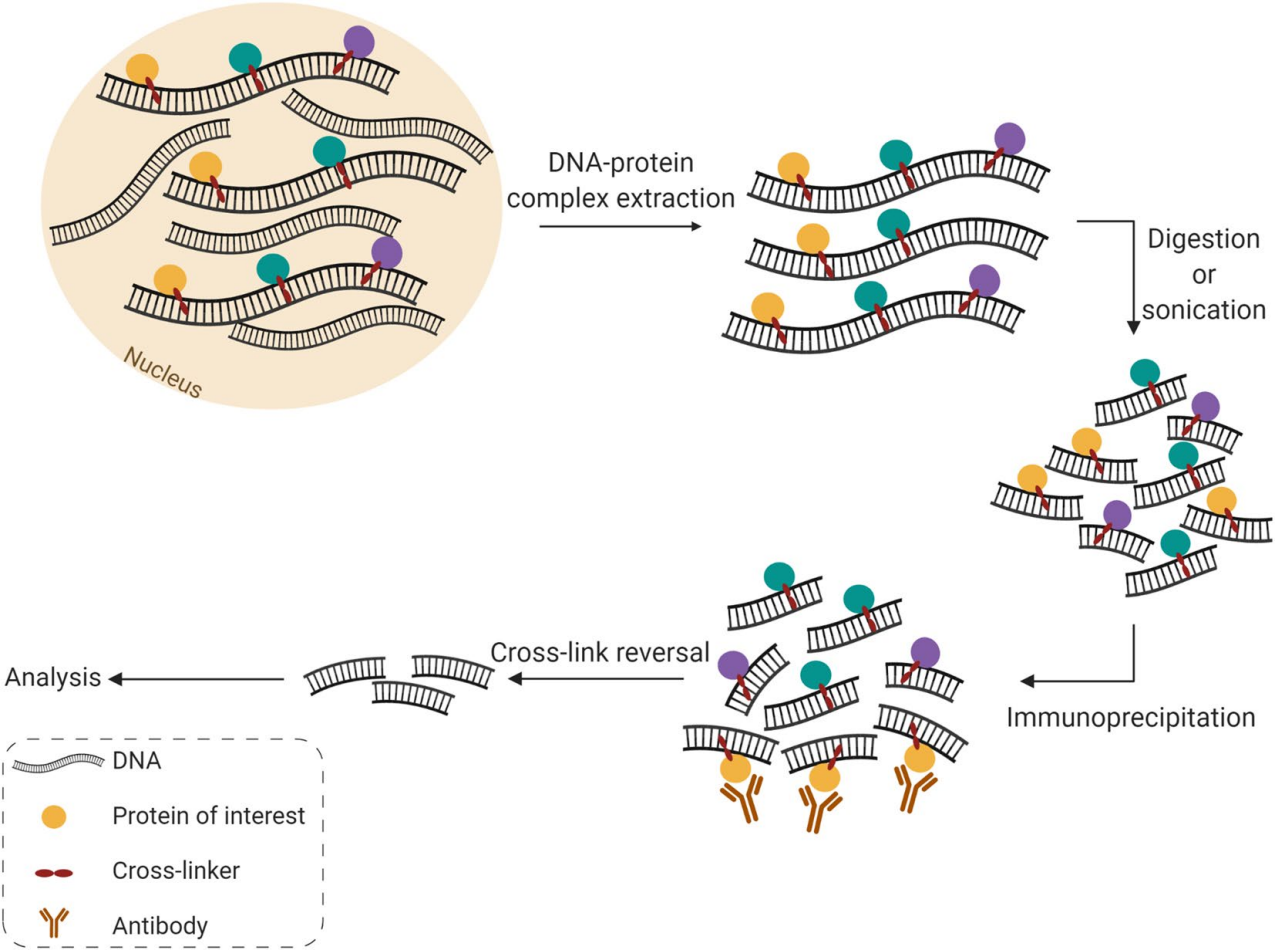
Illustration of the ChIP procedure. ChIP techniques usually start by the cross-link between DNA and proteins in a living cells using chemical cross-linkers or UV light. Subsquently, cross-linked chromatin is extracted and fragmented by digestion or sonication. The DNA–protein complexes under study are then immunoprecipitated using a specific antibody to the protein of interest and the obtained DNA is purified, released from the cross-link and analysed. Created with BioRender.com

A few years after the first EMSA publication, Gilmour and Lis [76] created the Chromatin ImmunoPrecipitation (ChIP) technique, which revolutionised the study of DNA–protein interactions. This assay starts by cross-linking DNA and proteins through a living cells treatment with chemical cross-linkers [42] or UV light [76]. Then, cross-linked chromatin is extracted and fragmented by digestion or sonication. Finally, the DNA–protein complexes of interest are precipitated, usually using a specific antibody to the protein under study, and the immunoprecipitated DNA is purified, released from the cross-link and analysed by different methods according to the purpose of the experiment, such as Southern blotting, PCR, qPCR, hybridisation to arrays or cloning, and sequencing [42] (Fig. 8). Some of the methods that complement ChIP are discussed below.

Blat and Kleckner [77] broadened the range of the ChIP technique and developed the ChIP-chip assay, which combines ChIP with microarray technology. In this method, after reverting the cross-link, the immunoprecipitated DNA is labelled and hybridised to a microarray that contains a group of DNA sequences of interest. The same process is performed with a DNA sample that was not precipitated as a control for variations in hybridisation intensity unrelated to the ChIP enrichment. Then, the relative binding of the protein under study to each sequence is calculated. While Blat and Kleckner [77] were investigating binding sites along yeast chromosome III, Ren et al. [78] performed a genome-wide study using a microarray that included all yeast intergenic sequences. Furthermore, the authors also combined both enriched and control DNA in the same microarray, but with different labels. Still, ChIP-chip presents several disadvantages. Firstly, amplification and hybridisation to probes of the immunoprecipitated fragments can introduce hybridisation noise signals from biased amplification. Also, the existence of different array designs and genome assemblies can lead to difficulties in comparing results from different groups.

In 2004, Impey et al. [79] developed a method combining ChIP with SAGE, which can be applied to genome scanning for TF binding site, and creating the ChIP-SAGE assay. According to this technique, after obtaining the DNA fragments immunoprecipitated during ChIP and performing several amplification and restriction reactions, ditags are joined in order to create a concatemer that is transformed into bacteria, amplified through replication and isolated. Finally, each 21 bp tag is sequenced and the respective recurrence is quantified. This technique comprehends the whole genome and does not require a priori acquaintance of sequences. Furthermore, the results obtained with these technique are measured more quantitatively, unlike ChIP-chip, that analyses spot intensities [79]. Nevertheless, this method involves several DNA amplification steps, which can introduce amplification bias. Moreover, although this technique helps in localising unique sites in the genome, it fails due to mapping ambiguity. Thus, ChIP-SAGE has a lower accuracy than ChIP-chip [80].

Two years later, Wei et al. [81] created a procedure quite similar to the previous one, where the immunoprecipitated fragments are cloned into a DNA library for propagation in bacteria. Then, the fragments are converted into paired-end ditags (PET), which are concatenated and cloned into a final plasmid for sequencing. These PET sequences are then mapped to the genome under study, with the possibility of originating a PET cluster when PETs from the same locus that contains the target binding site overlap [81]. This can lead to the prediction of new binding motifs present in the overlapping regions. Furthermore, this procedure does not introduce amplification bias. Thus, it improves the mapping accuracy of short-tags and increases the information content, relatively to the previous method. However, it also demands a large sequencing capacity [80].

Finally, the dawn of next generation sequencing (NGS) made it possible to decipher millions of DNA fragments quickly, simultaneously and efficiently. In 2007, Johnson et al. [82] combined ChIP with ultrahigh-throughput DNA sequencing, creating a simple and robust technique for global, unbiased examination of the binding sites, the ChIP-seq. In this procedure, after making the ChIP DNA’s ends blunt and ligating them with sequencing adaptors, limited PCR amplifications are performed. DNA fragments with selected sizes are amplified and sequenced in clusters using NGS technology. The sequences are then mapped to the genome of interest and regions with a high number of clusters of tag sequences are identified as ChIP enrichment sites. The non-specific sites are recognised, comparing the results with the ones obtained using a control DNA sequence, and removed [80].

In addition to the advantages of the previous two techniques, like the inexistence of hybridisation noise signals, the obtention of rigorous and quantifiable results and the genome coverage, the lower required amounts of ChIP DNA and, mainly, the higher base pair resolution are possibly the most notorious features of ChIP-seq. However, ChIP-seq also presents some disadvantages. The choice of the antibody used is crucial in ChIP-seq, as well as in all ChIP techniques. Even extremely specific antibodies may react positively with nuclear proteins different from the one under study [50]. Furthermore, some sequencing errors can still exist, though the improvements that have been made to the NGS methods. Moreover, insufficient reads can lead to a loss of sensitivity and specificity in spotting the enriched regions. A large amount of sequencing may be needed in order to acquire a sufficient number of tags throughout the genome and obtain accurate estimations. Nevertheless, the dominant drawback of ChIP-seq is its high cost. Still, as the cost of sequencing continues to decrease, ChIP-seq is expected to become the desired method concerning ChIP experiments in the near future [83].

Another problem associated with ChIP-seq is its precision, since the DNA molecules present in the libraries that result from standard ChIP-seq experiments have a length of approximately 200 bp. However, a protein usually binds only 6–20 bases. Furthermore, DNA contaminations are usual in these libraries, resulting from DNA that was not bound by the target protein and leading to systematic errors. In 2011, Rhee and Pugh [84] developed a new technique that eliminates this problem, ChIP-exo, according to which exonucleases digest free DNA fragments and link them to a fixed distance from the bound protein [75].

Other ChIP experiments have also been created, like Re-ChIP [85], according to which the binding to multiple proteins is tested for a single DNA sequence, and chromatin interaction studies [75]. The advantages, disadvantages and applications of each ChIP technique are present in Table 4.

**Table 4:** Advantages and disadvantages of ChIP techniques

Technique [References]	Technique		Applications	Case studies
	Pros	Cons		
ChIP [42]	In vivo method Enables observation of highly dynamic events	Requires a population of cells Cannot directly indicate functional significance	Predict the location of a bound protein in a particular cell type	Liu et al. [46]
ChIP-chip [42, 80]	High throughput Enables to calculate the relative binding affinity of the protein under study to each sequence	Hybridisation noise signals from biased amplification Difficulties in comparing results from different groups	Detect the presence of a specific protein throughout a large portion of the genome	
ChIP-SAGE [80]	The results obtained are measured more quantitatively	It suffers from mapping ambiguity	Predict the location of a specific protein throughout a large portion or the entire genome	
ChIP-PET [80]	The results obtained are measured more quantitatively Improves the mapping accuracy of short-tags and the information content	Demands a large sequencing capacity		
ChIP-seq [75, 80, 83]	Inexistence of hybridisation noise signals Obtention of rigorous and quantifiable results	Insufficient reads can lead to a loss of sensitivity and specificity in spotting the enriched regions High cost		
ChIP-exo [75]	Enables observation of highly dynamic events		Predict the location of more than one bound protein in a particular cell type	

## Case studies: recent uses and developments of techniques describing DNA–protein interactions

Although some of the referred techniques are not so used currently, many of them were applied recently, sometimes complementing more modern techniques, or with slight modifications and improvements, proving their significance to unravel some aspects of the nucleic acid–protein interaction.

For instance, not long ago, Prabu et al. [45] combined the filter binding assay with SELEX in order to identify potent aptamers that bind to the Human Pituitary Tumour Transforming Gene 1 protein (PTTG1). Prabu et al. [45] were able to detect 3 aptamers that showed high frequencies of appearance. The authors also used the filter binding assay to determine the equilibrium dissociation constant of each aptamer, assuming a 1:1 binding stoichiometry between the aptamer and protein, and concluded that the third aptamer presented the higher binding affinity towards the PTTG1 protein.

Trying to decipher how the phytochrome-interacting factor 4 (PIF4) negatively regulates the transcription of the *production* *of* *anthocyanin* *pigment1* (*PAP1*) gene in *Arabidopsis* *thaliana* seedlings, Liu et al. [46] performed a Y1H to discover whether PIF4 binds to the promoter of *PAP1*. The authors integrated *PAP1* promoter sequences fused to a resistant gene into Y1H Gold chromatin and identified three promoter sequences that interacted with PIF4, since the yeast strains generated presented high growth rates in media containing antibiotic. Furthermore, the authors also performed a ChIP-qPCR assay in order to test this interaction on Arabidopsis. After creating transgenic plants for the *35S:PIF4-HA* construct and using anti-HA antibody in the ChIP-qPCR assay, Liu et al. [46] concluded that PIF4 had a higher affinity for the *PAP1* binding site than the internal control (*Actin2*), suggesting that this factor could indeed bind to *PAP1* in vivo. Moreover, the authors also performed an EMSA using the binding protein and a sequence containing the binding site. Liu et al. [46] concluded that PIF4 binds to the *PAP1* gene promoter via the binding motif, since the electrophoretic lane containing the promoter sequence and PIF4 presented a delayed band, analogously to the lane containing the same parts together with a mutated competitor sequence and oppositely to what happened when using a mutated target sequence or when adding a wild-type competitor fragment. Finally, the authors also performed a transient transcriptional expression analysis using Arabidopsis protoplasts, in order to understand if the interaction between the two parts would lead to the down-regulation of *PAP1* expression. After introducing constructs containing the luciferase gene regulated by wild-type or mutated *PAP1* promoter sequences into different protoplasts together with constructs containing the PIF4 gene, Liu et al. [46] observed that the activation of the reporter gene was much lower in protoplasts containing constructs with the wild-type promoter and the PIF4 gene than in protoplasts with constructs containing the mutated promoter and the PIF4 gene. Thus, the authors concluded that PIF4 has a negative regulatory effect on the *PAP1* promoter function.

In order to identify the proteins that link to the *Solanum* *lycopersicum* *LeSPL-CNR* (*squamosa* *promoter* *binding* *protein-like-colourless* *non-ripening*) gene, Wang et al. [47] conducted a South-Western Blotting using a digoxigenin (DIG)-labelled 286 bp probe of this gene and a protein extract from tomato fruits. After performing a two-dimensional electrophoresis, the authors transferred the proteins to a PVDF membrane containing the labelled probes and identified 13 tomato proteins with known or predicted functions that linked to the fragment tested.

Recently, Manosas et al. [55] developed a high-throughput mechanism that can be applied to single molecule footprinting of small and large DNA ligands. Single molecule footprinting consists in applying a mechanical force with magnetic tweezers at the opposite ends of a DNA hairpin in order to disrupt the base-pairs, unzipping the DNA cooperatively in one step. However, the addition of ligands, such as proteins, leads to a multi-step untangle of the DNA caused by the ligand binding, enabling to determine the ligand sequence specificity. Through the measurement of the binding lifetimes at different forces, binding kinetics can be studied. The authors designed hairpin DNA molecules that allow to perform these measurements with a flat free energy landscape and test several binding sequences in a single assay, widening the repertoire of DNA footprinting assays.

Song et al. [74] used a biosensor surface in order to kinetically analyse the DNA–protein interactions of wild-type/mutant p53 proteins through real-time monitoring of the localised surface plasmon resonance shift. These case studies are referred in Tables 1, 2, 3 and 4.

## Conclusion

Even though there are several and different techniques to describe, analyse and compare DNA–protein interactions, they can be organised into separate sections, according to the respective purpose. Filter binding assay and EMSA are easy in vitro methods that rapidly identify nucleic acid–protein binding interactions, being the procedures of choice in many functional studies that aim to confirm the gene targets of a given TF. Together with SDS-PAGE, EMSA can help in the identification of the proteins that bind to a certain sequence when using crude protein extracts. Cross-linking can also be used with this objective, especially when dealing with DNA–protein complexes that involve more than one protein that can be indirectly linked to the DNA. In vivo methods, like Y1H and PTA, can also be applied when analysing if a TF binds to a given sequence. These approaches provide reliable results since they recreate similar environments to the ones where the complex is formed. When trying to discover the DNA-binding sites localisation, DNA-footprinting is indeed an easier, faster and relatively reliable approach, which also allows to investigate the impact of each nucleotide on the binding affinity. Furthermore, in vivo footprinting methods are also available. Nevertheless, techniques involving base analogues and base-site selection are more expensive, but confer a higher precision and are quite helpful when trying to identify aptamers that link to a given protein, as in the example described in the previous section. Quantifying the kinetics and affinity of a given DNA–protein interaction can be useful, mainly when comparing more than one interaction. Once again, filter binding assay and EMSA are useful and easy methods in this subject. SPR and methods applied to the proteins study that can also be used in the analysis of nucleic acid–protein interactions, like circular dichroism and fluorescence spectroscopy, are quite sensitive and direct approaches. However, these procedures involve specific instruments and technology. Finally, considering genome-wide studies, ChIP-seq is the desired method, given the coverage and resolution of the technique. Nevertheless, the costs related to NGS remain a barrier to the use of this technique. In conclusion, although some experiments are easier, less expensive and faster than others, when envisioning a DNA–protein interaction study several aspects cannot be disregarded, since several useful and available methods can confer more precise and accurate results in a simpler way than one may think.

## Data Availability

Not applicable.

## Abbreviations

AD: Activation domain
CAP: Catabolite gene activator protein
CE-LIF: Capillary electrophoresis–laser-induced fluorescence
ChIP: Chromatin immunoprecipitation
DIG: Digoxigenin
EMSA: Electrophoretic mobility shift assay
*LeSPL-CNR*: *Solanum* *lycopersicum* *squamosa* *promoter* *binding* *protein-like-colourless* *non-ripening*
NGS: Next generation sequencing
*PAP1*: *Production* *of* *anthocyanin* *pigment1*
PET: Paired-end ditags
PIF4: Phytochrome-interacting factor 4
PTA: Protoplast transactivation assay
PTTG1: Human pituitary tumour transforming gene 1 protein
PVDF: Polyvinylidene difluoride
RU: Resonance units
SAGE: Serial analysis of gene expression
SDS-PAGE: Sodium dodecyl sulphate-polyacrylamide gel electrophoresis
SELEX: Systematic evolution of ligands by exponential enrichment
SPR: Surface plasmon resonance
TEAMP: Transient expression in arabidopsis mesophyll protoplast
TF: Transcription factor
*WOX8*: *Wuschel* *related* *homeobox* *8*
Y1H: Yeast one-hybrid
